# *HIC1* and *RassF1A* Methylation Attenuates Tubulin Expression and Cell Stiffness in Cancer

**DOI:** 10.3390/ijms19102884

**Published:** 2018-09-22

**Authors:** Chih-Cheng Chen, Bo-Ching He, Yao-Li Chen, Kuan-Der Lee, Chun-Hsin Tung, Chia-Chen Hsu, Ping-Yi Lin, Pei-Yi Chu, Yu-Wei Leu, Wei-En Fu, Shu-Huei Hsiao

**Affiliations:** 1 Department of Hematology and Oncology, Chang Gung Memorial Hospital, Chiayi 61363, Taiwan; ccchen1968@gmail.com (C.-C.C.); loofahhsu@gmail.com (C.-C.H.); 2 Chang Gung Institute of Technology, Taoyuan 33302, Taiwan; 3 Nano and Mechanical Measurement Laboratory, Center for Measurement Standards, Industrial Technology Research Institute, HsinChu 31057, Taiwan; hopc@itri.org.tw (B.-C.H.); weienfu@itri.org.tw (W.-E.F.); 4 Transplant Medicine & Surgery Research Centre, Changhua Christian Hospital, Changhua 50006, Taiwan; 31560@cch.org.tw (Y.-L.C.); 69221@cch.org.tw (P.-Y.L.); 5 School of Medicine, Kaohsiung Medical University, Kaohsiung 80708, Taiwan; 6 Division of Hematology and Oncology, Department of Medicine, Taipei Medical University Hospital, Taipei 11050, Taiwan; kdlee1964@gmail.com; 7 Human Epigenomics Center, Department of Life Science, Institute of Molecular Biology and Institute of Biomedical Science, National Chung Cheng University, Chiayi 62102, Taiwan; chun.hsin.tung@gmail.com (C.-H.T.); bioywl@ccu.edu.tw (Y.-W.L.); 8 Department of Pathology, Show Chwan Memorial Hospital, Changhua 50008, Taiwan; chu.peiyi@msa.hinet.net

**Keywords:** DNA methylation, AFM, stiffness, cancer

## Abstract

Cell stiffness is a potential biomarker for monitoring cellular transformation, metastasis, and drug resistance development. Environmental factors relayed into the cell may result in formation of inheritable markers (e.g., DNA methylation), which provide selectable advantages (e.g., tumor development-favoring changes in cell stiffness). We previously demonstrated that targeted methylation of two tumor suppressor genes, hypermethylated in cancer 1 (*HIC1*) and Ras-association domain family member 1A (*RassF1A*), transformed mesenchymal stem cells (MSCs). Here, transformation-associated cytoskeleton and cell stiffness changes were evaluated. Atomic force microscopy (AFM) was used to detect cell stiffness, and immunostaining was used to measure cytoskeleton expression and distribution in cultured cells as well as in vivo. *HIC1* and *RassF1A* methylation (*me_HR*)-transformed MSCs developed into tumors that clonally expanded in vivo. In *me_HR*-transformed MSCs, cell stiffness was lost, tubulin expression decreased, and F-actin was disorganized; DNA methylation inhibitor treatment suppressed their tumor progression, but did not fully restore their F-actin organization and stiffness. Thus, *me_HR*-induced cell transformation was accompanied by the loss of cellular stiffness, suggesting that somatic epigenetic changes provide inheritable selection markers during tumor propagation, but inhibition of oncogenic aberrant DNA methylation cannot restore cellular stiffness fully. Therefore, cell stiffness is a candidate biomarker for cells’ physiological status.

## 1. Introduction

Environmental factors may reform the cellular epigenome [1], physiology, and physical appearance, including cell stiffness. The reformed appearance might affect cellular transformation and metastasis [2]. Signals, such as estrogen signals [3] and signals from other cocultured cells [4], are relayed into cells through signaling pathways and induce epigenetic changes within the target genes of that particular signal. DNA methylation is an epigenetic change inherited in somatic lineages [5] that dominantly silences the associated genes [6,7]. Accumulated changes in epigenetics [8], gene expression, and cell physiology elicit phenotypic variation in cells, such that the affected cells can be further selected for transformation by their environment [2]. Cells that survive and adapt can be expanded clonally.

The aforementioned noteworthy hypothesis has not been investigated thoroughly. As described, environmental interferences within the epigenome have been widely documented. Abnormal epigenetic modifications can be generated at low rates and accumulate in somatic cells after DNA damage and repair [9]. Two lines of evidence demonstrate that abnormal DNA methylation is sufficient to transform a cell: (1) Global hypomethylation in mice can induce cancer [10,11]. (2) Mesenchymal stem cells (MSCs) can be transformed into cancer stem cell (CSC)-like cells after specific methylation of two tumor suppressor genes, hypermethylated in cancer 1 (*HIC1*, NM_001107021) and Ras-association domain family member 1A (*RassF1A*, NM_001037555.1) [12]. To completely understand these modifications, determining how abnormal methylated cells gain their competitive phenotypic edge during transformation is necessary. 

Cell plasticity, including loss of stiffness that can be measured using techniques such as atomic force microscopy (AFM) [13,14], represents a phenotypic edge during tumor development [15]. Compared with normal cells, tumor cells often compete for or reside outside the usual niches and demonstrate abnormal cell morphology histologically [16,17,18,19]. Out-of-shape or loss of stiffness originates from loss of internal cytoskeleton supports [20,21,22,23] and allows cancer cells to migrate and adapt to other tissues [24,25]. Abnormal expression or dysregulated cytoskeleton assembly is associated with tumorigenesis. Drugs, such as Taxol, target members of the cytoskeleton, including tubulin, and are used for cancer therapy [26]. 

*RassF1A* and its effectors were found to control microtubule organization [27], and methylation-silenced *RassF1A* and the subsequent cytoskeleton disorganization contribute to the development of drug resistance in cancer [28]. Therefore, *HIC1* and *RassF1A* methylation (*me_HR*)-transformed cells may lose normal microtubule expression and organization, including stiffness, and adapt and express different markers. Such methylation-induced tumoral properties could then be reversed using demethylation agents.

We previously reported that *me_HR*-transformed MSCs are resistant to drugs, such as Taxol [12], but the resistance is reversed after treatment with 5-aza-2′-deoxycytidine (5-Aza), a DNA demethylation agent [29]. In the current study, we measured loss of cellular stiffness through AFM and detected corresponding tubulin downregulation in cultured cells and inoculated animal models during transformation through immunostaining. Tubulin expression was restored with 5-Aza, but not the overall tubulin organization. Finally, abnormal *HIC1* and *RassF1A* methylation was present in 96 liver cancer samples. Aberrant gene expression, particularly decreased *RassF1A* expression, was associated with tubulin downregulation in selected samples. Therefore, the association among methylation, transformation, and cell plasticity changes was strengthened further.

## 2. Results

### 2.1. HIC1 and RassF1A Methylation Transforms MSCs

#### 2.1.1. Repeated HIC1 and RassF1A Methylation Can Transform MSCs into CSC-Like Cells

**Figure 1:**
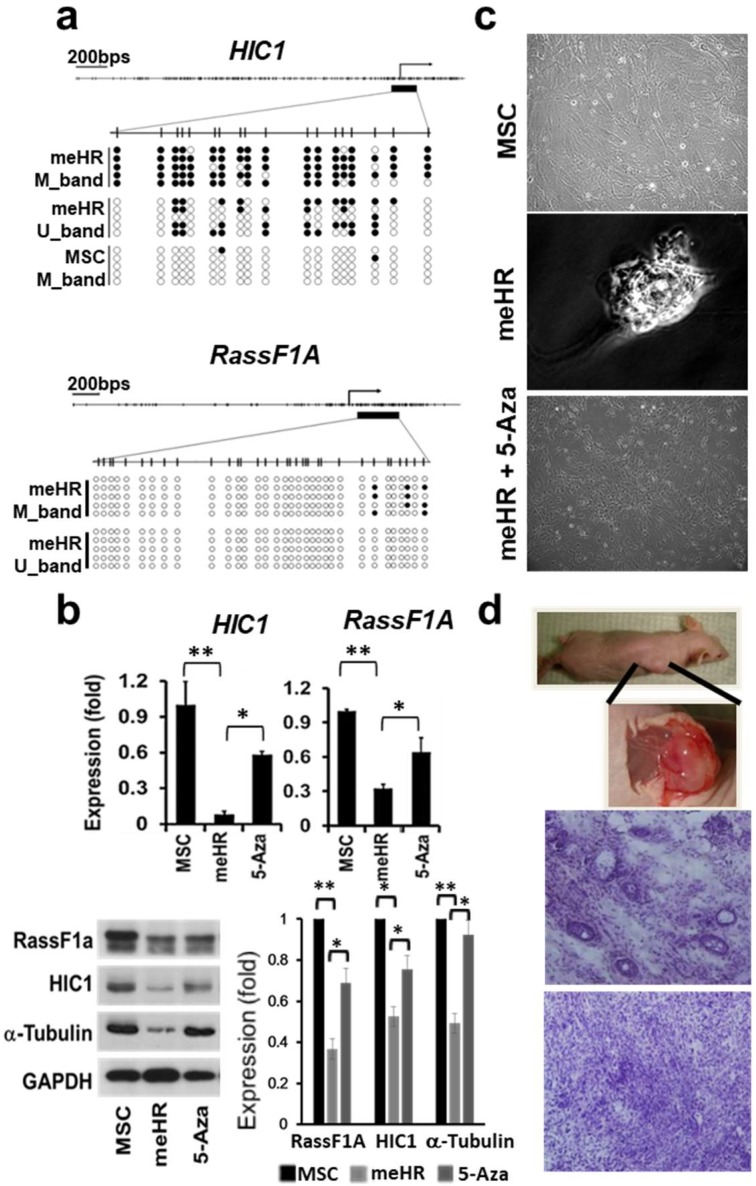
Targeted HIC1 and RassF1A methylation transforms mesenchymal stem cells (MSCs). (**a**) Validation of targeted HIC1 and RassF1A methylation. Targeted DNA methylation in HIC1 and RassF1A was validated by sequencing methylation-specific polymerase chain reaction (PCR) products and pyrosequencing, respectively. Vertical short bars within physical maps indicated the CpG loci and their methylation percentages were revealed by the filled density in circles. U-band: methylation-specific polymerase chain reaction amplified by unmethylated primers; M-band: methylation specific PCR (MSP) amplified by methylated primers. (**b**) Targeted HIC1 and RassF1A methylation reduced expression, reversed by 5-aza-2′-deoxycytidine (5-Aza) treatment. Targeted HIC1 and RassF1A methylation reduced their respective gene expression as detected through semi-quantitative reverse transcription PCR (top) and Western blotting (lower two, right panel indicates the quantitative summaries of the repeated Western blots on the left) experiments in me_HR. The methylation-silenced gene expression was reversed in the me_HR-transformed MSCs after 5 days using 5-Aza treatment (5 μM). (**c**) Loss of contact inhibition in me_HR-transformed MSCs. MSCs possessed contact inhibition phenotype (top, with 200 folds of magnification), whereas me_HR-transformed MSCs lost contact inhibition in low cell numbers (center, with 400 times magnification). The loss of contact inhibition was reversed after me_HR-transformed MSCs were treated with 5 μM 5-Aza for 5 days (lower, 200 times magnification). (**d**) me_HR-transformed MSCs grew into tumors after inoculation into nude mice. At a low cell number, me_HR-transformed MSCs could develop into tumors after inoculation into nude mice (top, 200 times magnification). Tumors that developed were surgically excised and examined through hematoxylin and eosin staining (lower, 200 times magnification). The quantified data among treatments were compared using paired *t* test (* *p* < 0.05; ** *p* < 0.01; *n* = 3).

HIC1- and RassF1A-targeted methylation was demonstrated by sequencing the products of HIC1 methylation-specific polymerase chain reaction (PCR) and RassF1A bisulfite pyrosequencing (Figure 1a). Increased DNA methylation within their promoters was associated with decrease in the expression of HIC1 and RassF1A (Figure 1b). The methylation-silenced expression was reversed after 5-Aza treatment, thus proving that silencing was caused by the DNA methylation. Along with transformation and drug resistance development (Figure S1) [12], tubulin expression decreased, indicating possible cell stiffness loss. Along with changes at the epigenetic level, cell morphology changes occurred. The transformed cells lost their contact inhibition in culture (Figure 1c, center), and the loss of contact inhibition phenotype could be reversed with 5-Aza treatment. The inoculated, transformed MSCs developed tumors in immunodeficient (nude) mice (*n* = 13, Figure 1d) at low cell numbers, but the control cells did not. Therefore, changes in HIC1 and RassF1A methylation can transform MSCs.

#### 2.1.2. Clonally Expanded me_HR-Transformed MSCs

After transformation and inoculation into nude mice, the seeded me_HR-transformed cells began expanding and clonally expressing different cell surface markers. As shown in Figure S2a,b (center panel, green), previously CD133-nonexpressing MSCs began expressing possible stem cell markers [30,31] in the tumor mass. Individual or groups of the CD133-expressing MSCs were surrounded by a cell mass expressing different cell surface and tumor markers, such as cytokeratin (Figure S2a) and NSE (Figure S2b). Although most cells still expressed MSC markers, such as vimentin (Figure S2c, top left), some cells clonally expressed different markers, such as epithelial markers. These clonally expressed markers indicated further tumoral evolution of me_HR-transformed MSCs and revealed their plasticity.

### 2.2. Loss of Stiffness Correlates with the Loss of Tubulin Expression in me_HR-Transformed MSCs

**Figure 2:**
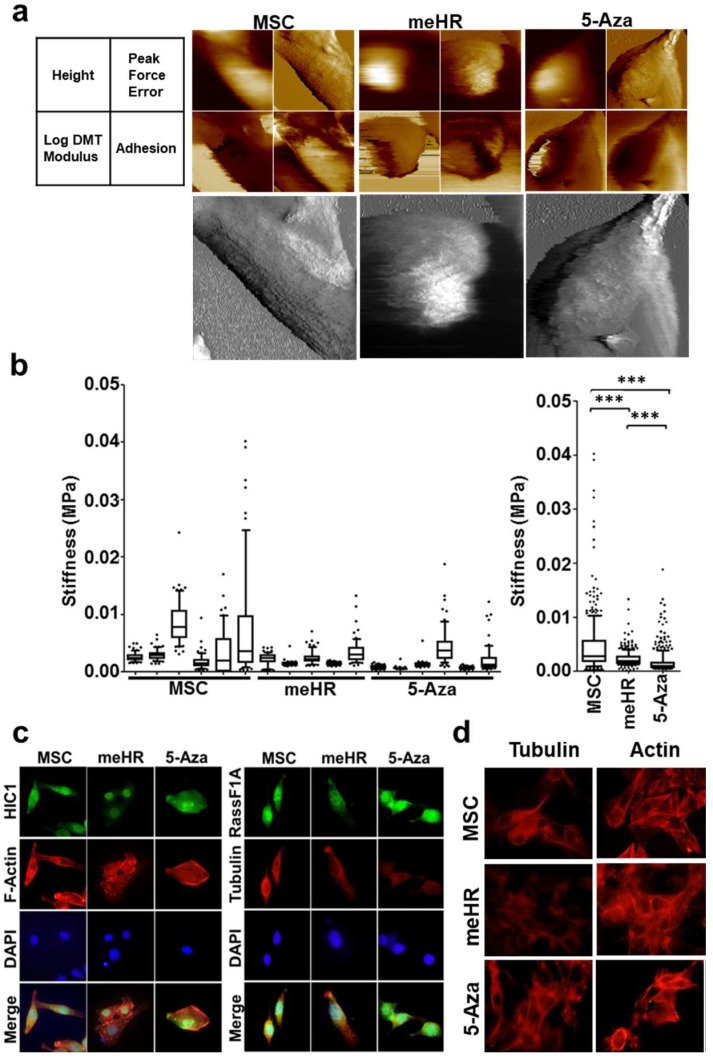
Loss of cell stiffness in me_HR-transformed mesenchymal stem cells (MSCs) is correlated with loss of tubulin expression, and it can be reversed by 5-aza-2′-deoxycytidine (5-Aza) treatment. (**a**) Cell surface morphology and cell stiffness of MSCs, me_HR-transformed MSCs, and 5-Aza-treated me_HR-transformed MSCs were measured using atomic force microscopy (AFM) as described. Four representative derived moduli of the AFM measurements are illustrated. The peak force error modulus is shown in black and white and is enlarged in the lower panels to highlight the surface spines (with 400 times magnification). (**b**) Loss of cell stiffness in me_HR-transformed MSCs. Representative cell stiffness measurements of at least five individual, me_HR-transformed, and 5-Aza-treated me_HR-transformed MSCs are box-plotted on the left (measurement was repeated twice, *n* = 3, *** *p* < 0.001). Overall stiffness distribution is plotted on the right. (**c**) Immunostaining of MSCs, me_HR-transformed MSCs, and 5-Aza-treated me_HR-transformed MSCs. MSCs, me_HR-transformed MSCs, and 5-Aza-treated me_HR-transformed MSCs were seeded in four-well chamber slides, and indicated antibodies were used to detect expression (with 400 times magnification). (**d**) Detection of tubulin and β-actin expression in MSCs, me_HR-transformed MSCs, and 5-Aza-treated me_HR-transformed MSCs (with 400 times magnification). Enlarged immunostaining data are illustrated in Figure S3. DMT: Derjaguin–Muller–Toporov.

**Figure 3:**
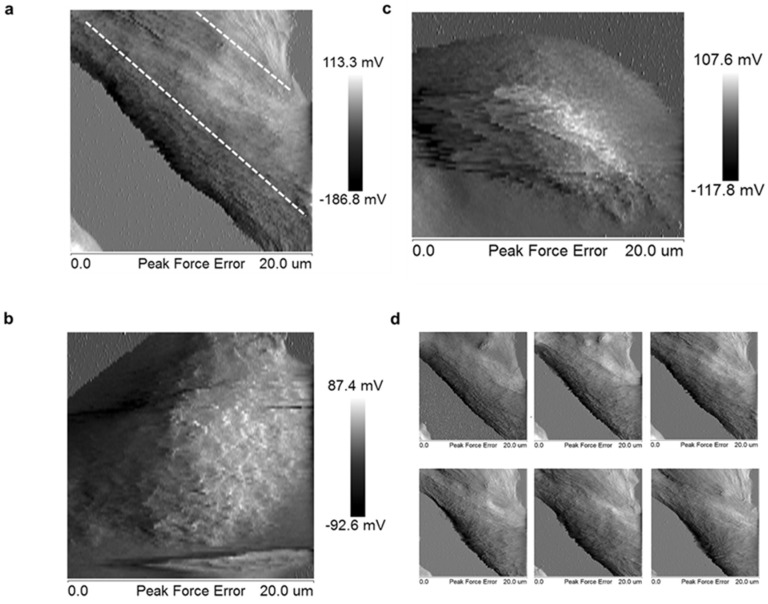
Loss of organized cell surface structure in me_HR-transformed mesenchymal stem cells (MSCs). Cell surface morphology of MSCs (**a**), me_HR-transformed MSCs (**b**), and 5-aza-2′-deoxycytidine-treated me_HR-transformed MSCs (**c**) were measured through AFM. The peak force error moduli are shown in black and white after being transformed using NIH Image and enlarged at the same proportion to highlight the surface spines. Dashed lines indicate the orientation of the organized cell surface structures. (**d**) Sequential peak force errors of the same MSCs cells were correlated with cell movement.

Loss of cell stiffness is the physical evidence of cell plasticity gain and can be detected using AFM. Cell stiffness was measured through AFM (Figure 2a) and normal cells, such as MSCs, had a wide range of stiffness compared with me_HR-transformed MSCs (Figure 2b). Treatment with 5-Aza partially restored the stiffness (Figure 2b). We also observed that organized spines were present on the surfaces of the MSCs, which were disorganized on the surface of untreated and 5-Aza-treated me_HR-transformed MSCs (Figure 2a, lower panels). Peak force error images were further transformed using NIH Image into black and white to visualize the cell surface structure in higher contrast (Figure 3). Figure 3a–c represents the transformed images from MSCs, me_HR-transformed MSCs, and 5-Aza-treated me_HR-transformed MSCs, respectively. The directions of the spines are projected as two dashed lines in Figure 3a,c, but no obvious directions were observed in the me_HR-transformed MSCs (Figure 3b). The organized spine directions correlated with the cell movement during division as demonstrated by sequential AFM measurement (Figure 3d and the compiled gif movie in Supplementary File 2). These structures closely correlated with the gain or loss of stiffness. F-actin structures were disorganized in untreated and 5-Aza-treated me_HR-transformed MSCs compared with normal MSCs (Figure 2c, left). Tubulin expression decreased in the me_HR-transformed MSCs but restored in 5-Aza-treated me_HR-transformed MSCs (Figure 2c, right panels). Tubulin expression loss and F-actin disorganization were highly evident in me_HR-transformed MSCs, as shown in Figure 2d.

### 2.3. Reduced Tubulin Expression in me_HR Tumors

**Figure 4:**
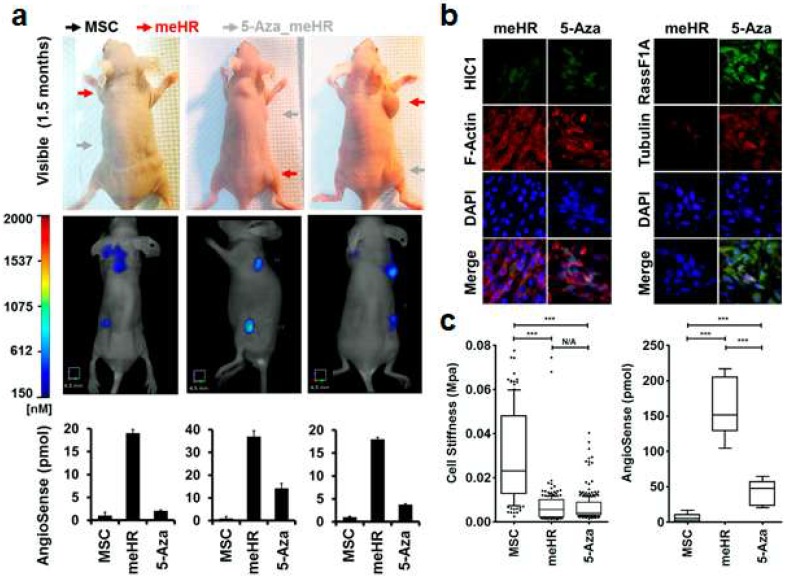
Targeted HIC1 and RassF1A methylation-induced loss of tubulin expression in vivo. Mesenchymal stem cells (MSCs), me_HR-transformed MSCs, and 5-aza-2′-deoxycytidine (5-Aza)-treated me_HR-transformed MSCs were inoculated into nude mice to observe their tumoral development in vivo. (**a**) Inoculated me_HR cell tumors were reversed by 5-Aza treatment before inoculation. Arrows indicate the subcutaneous inoculation sites. The tumor mass was tracked using the florescent dye AngioSense, attached to the tumor. It allowed emitted light to be visualized and quantified using a fluorescence tomography, FMT 4000 machine (in vivo imaging system, Perkin Elmer, Billerica, MA, USA). Inoculation with me_HR-transformed MSCs (red arrows) led to large tumor masses, whereas that with 5-Aza-treated me_HR-transformed MSCs did not (gray arrows). Inoculated MSCs (black arrows) did not grow into tumor masses or exhibit background readings because the dye was injected through the tail vein. (**b**) Reduced tubulin expression in me_HR-induced tumors in vivo. Tumors from mice in (**a**) were surgically removed, cryosectioned, and immunostained using the indicated antibodies. HIC1, RassF1A, and tubulin expression were reduced in me_HR-transformed MSC-inoculated tumors but upregulated in smaller tumors from 5-Aza-treated me_HR-transformed MSC-inoculated mice (with 200× magnification). (**c**) Correlation between the loss of stiffness and increased tumoral growth in vivo. The loss of stiffness (left) and increased tumoral growth (right) in me_HR-transformed MSC-induced tumors are plotted side by side for comparison (*** *p* < 0.001).

Tubulin expression in me_HR-transformed MSCs decreased in vivo. MSCs, me_HR-transformed MSCs, and 5-Aza-treated me_HR-transformed MSCs were inoculated into nude mice in different regions of the same mice (arrows in Figure 4a). The me_HR-transformed MSCs developed into tumors, as tracked using the tumor marker AngioSense dye in vivo, whereas the MSCs did not. The 5-Aza-treated me_HR-transformed MSCs also developed into tumors but were slower growing and exhibited decreased mass compared with the me_HR-transformed MSCs (Figure 4a). The tumors resulting from the untreated and 5-Aza-treated me_HR-transformed MSCs were surgically removed and sectioned. Next, HIC1, RassF1A, F-actin, and tubulin expression were evaluated through immunostaining, and noted that HIC1, RassF1A, and tubulin expression were greatly reduced in the me_HR tumors, but their expression was restored after 5-Aza treatment and then inoculated (Figure 4b). The changed DNA methylation states also changed the cellular stiffness, tumor development in vivo (Figure 4c), and sustained tubulin expression loss in vivo. These correlated sequential events indicated that concurrent HIC1 and RassF1A methylation potentially plays a role in tumor expansion.

### 2.4. Tubulin Expression Correlates with RassF1A Expression in Liver Cancer

**Figure 5:**
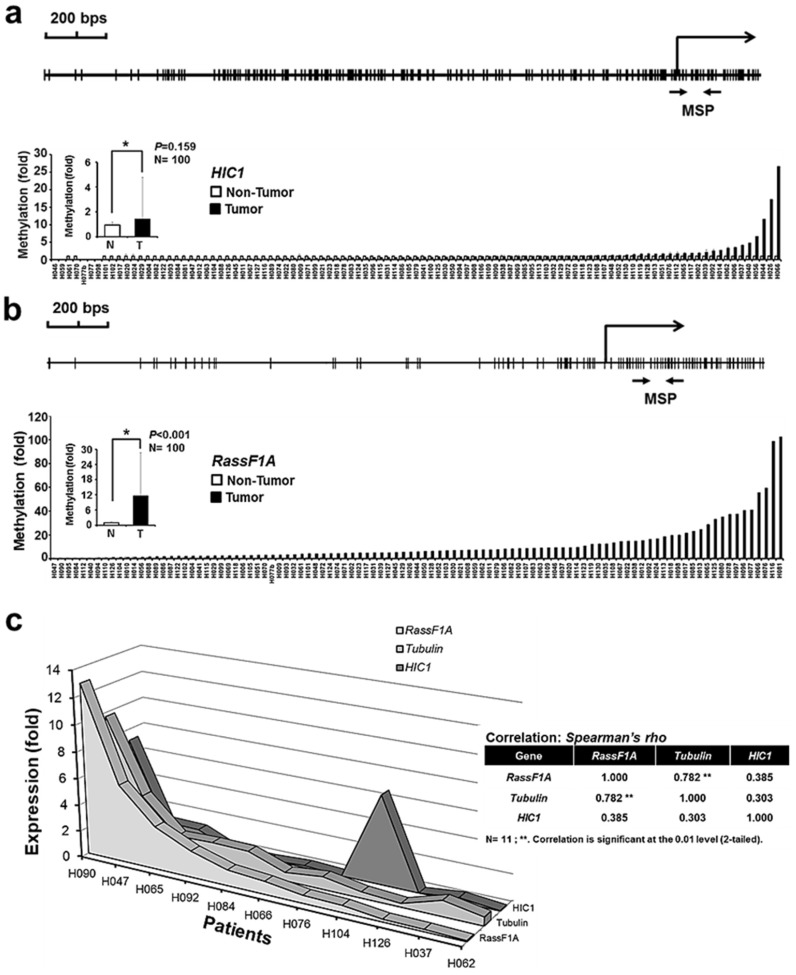
Abnormal HIC1 and RassF1A methylation and the associated loss of tubulin expression are observed in human liver cancers. (**a**) HIC1 and (**b**) RassF1A methylation states were detected in 96 liver cancer samples. The filled histogram indicates the methylation states from the tumor tissue, whereas the open histogram shows the methylation states from the adjacent normal tissue. (**c**) Tubulin expression was associated with RassF1A expression. Total RNAs from five pairs of hypomethylated and six pairs of hypermethylated liver cancer were isolated, and qRT-PCR was performed to examine HIC1, RassF1A, and tubulin expression. Spearman’s correlation analyses were used to identify a significant correlation between RassF1A and tubulin expression.

We next determined whether abnormal HIC1 and RassF1A methylation and the corresponding downregulation in tubulin expression occurred in human liver cancer. HIC1 and RassF1A methylation states were examined in 96 liver cancer sample pairs. We noted that the tumor regions were hypermethylated compared with adjacent normal tissue (Figure 5a,b). Correlation between the HIC1 or RassF1A methylation states and the liver cancer progression (also other clinical-pathological facts) are summarized in Supplementary Table S3 and S4 respectively. Total RNAs from the top six pairs of hypermethylated samples and top five hypomethylated pairs of samples were isolated; we further evaluated HIC1, RassF1A, and tubulin expression through semi-quantitative reverse transcription PCR (qRT-PCR; Figure 5c). We noted that changes in tubulin expression were closely correlated with changes in RassF1A expression (Figure 5c). Thus, abnormal methylation in the HIC1 and RassF1A loci might affect tubulin expression in vivo.

## 3. Discussion

### 3.1. DNA Methylation Is an Inheritable Tumor Marker

Environmental changes can be transmitted into cells and memorized as DNA methylation, which further shapes gene expression and cellular physiology in cells [8,32]. These lineage-inherited expression differences can be selected and thus the cells can transform and evolve further [1]. Inherited cell morphological changes include changes in cellular plasticity, such as cell identity and cell stiffness, critical for tumor mass development, developed tumor metastasis, and drug resistance development [2]. Each step of these morphological changes originates from somatic inheritable markers, such as genetic or epigenetic modifications, and these markers can be tracked to record tumor development. Recapitulation of these tumor development steps ensures the casual association between molecular and physical changes and establishes epigenetic changes as biomarkers. For instance, if targeted methylation of certain genes alone can transform a cell and if changes in cell physiology are inevitable, then methylation changes and physiological modifications are candidate tumor detection biomarkers.

### 3.2. Abnormal HIC1 and RassF1A Methylation Identified in Cancers

Tumor suppressor genes *HIC1* and *RassF1A* are hypermethylated in various cancers [33,34]. Whether the abnormality in DNA methylation can transform normal MSCs until concurrent targeted HIC1 and RassF1A methylation is demonstrated into a somatic CSC-like MSCs remains unknown [12,35]. HIC1 is a transcriptional factor that interacts with different signaling pathways and may be responsible for the maintenance of genome stability [36,37]. However, RassF1A controls cell cycle progression as well as cell death through several effector genes in different pathways [33,38,39]. Concurrent methylation of both loci only demonstrated that methylation abnormality can transform a cell but cannot prove that it occurs during tumorigenesis in patients or demonstrate how the cells are transformed. Further understanding regarding how inheritable methylation abnormality leads to changes in cell physiology, such a cell plasticity, is required. AFM-assisted cell stiffness detection could aid the discovery of the transformation mechanism.

### 3.3. Application of AFM in Cancer Research

AFM detects the cell and molecular level differences in human samples [40,41,42]. AFM can detect single-digit methylation changes along a stretch of DNA and different variations in sequences [43,44,45]. The basic interactions between molecules comprising the epigenome, including DNA methylation, were noted. AFM can detect subtle changes, such as cell stiffness in soft tissues and cells [41], including tumors [46]. It was hypothesized that the changes in cell stiffness are necessary for cancer development and progression. In cancer detection, for discovering the possible molecular mechanisms underlying the changes in cell stiffness, AFM might be a helpful tool.

### 3.4. Loss of Stiffness in me_HR-Transformed MSCs 

*HIC1* and *RassF1A* methylation transformed a somatic stem cell, MSC, into CSC-like cells, *me_HR*-transformed MSCs [12]. Here, we demonstrated that the cellular plasticity of the transformed cells changed as well. The *me_HR*-transformed MSCs possessed the original mesenchymal marker, vimentin, but started expressing the commonly observed stem cell marker CD133 (Figure S2) in the tumor mass that developed. Clones of the cells from the dissected *me_HR* tumor mass expressed different tumor markers, some of which were epithelial. Diverse marker expression indicated cell-fate transition and diversion (Figure S2). A direct measurement of cell stiffness through AFM revealed the loss of stiffness in the *me_HR*-transformed MSCs (Figure 2 and Figure 3). The loss of stiffness could be reversed using a methylation inhibitor, indicating that it was caused by DNA methylation (Figure 2 and Figure 3). During the measurements, the outer organized spine-like structures (Figure 3) were lost on *me_HR* transformation but were partially restored by 5-Aza treatment. This outer phenotype correlated with an internal loss of tubulin expression and disorganized actin distribution (Figure 4 and Figure S3), both of which were partially restored by 5-Aza treatment as well. These sequential molecular and phenotypic changes recapitulated the previously fragmented observations regarding tumorigenesis.

### 3.5. Correlation between Stiffness Loss and Drug Resistance Development in Cancer

In a previous study, we found that *me_HR*-transformed MSCs became drug-resistant and the drug-resistant phenotype could be reversed by 5-Aza treatment [12]. In other reports, the *RassF1A* promoter was often hypermethylated in several cancers [47]. In addition, *RassF1A* and its effectors can regulate tubulin expression. A report indicated that YAP, a *RassF1A* signaling pathway component, can model its dependent matrix and is required for cancer-associated fibroblast generation and maintenance [24,25]. Because tubulin is a target of anticancer drugs, such as Taxol, downregulated tubulin expression may increase tumor cell drug resistance. Therefore, hypermethylation of *RassF1A* or its associated pathway would decrease tubulin expression, subsequently increasing tumor drug resistance. This hypothesis was validated in *me_HR* tumor cells; the correlated expression of *RassF1A* and tubulin was observed in liver cancer samples.

### 3.6. Cell Stiffness as Another Biomarker

Abnormal DNA methylation [32] and cell stiffness [46] may be biomarkers of cancer development. Study findings have indicated that each of these markers has clinical potential. The data reported here have linked these biomarkers and imply that they might be combined to increase clinical accuracy. Conversely, demethylation agents might be able to reduce drug resistance in cancer if its development is originally methylation-dependent. Examination of *HIC1* and *RassF1A* methylation or tubulin expression might therefore help predict the best cancer treatment course.

## 4. Materials and Methods 

### 4.1. Cell Culture 

MSCs and their methylation-transformed counterparts (*me_HR*) were cultured in MEM-α (Invitrogen, Carlsbad, CA, USA) supplemented with 20% fetal calf serum, 100 mg/mL penicillin/streptomycin (Invitrogen), and 2 mM l-glutamine (Invitrogen). Cells were cultured in incubators supplied with 5% CO_2_/95% O_2_ at 37 °C.

### 4.2. 5-Aza Treatment

Cells were treated with 5 μM 5-Aza or an equal final volume of DMSO for 5 consecutive days.

### 4.3. Western Blotting

Total protein was harvested from cells using cell lysis buffer and the protein concentration was quantified. Proteins were denatured and separated using 10% SDS-PAGE (25 μg protein per lane). After trans-blotting and blocking with 5% skin milk, blots were incubated with RassF1A (eBioscience, Thermo Fisher Scientific, Waltham, MA, USA), HIC1 (Abnova, Walnut, CA, USA), Tubulin (GeneTex, Irvine, CA, USA), and GAPDH (Millipore, Burlington, MA, USA) antibodies, followed by hybridization with peroxidase-coupled secondary antibodies and detection using the ECL system (Millipore).

### 4.4. Semi-Quantitative RT-PCR, qRT-PCR

Total RNA isolation, first-strand cDNA synthesis, and detection of transcripts were performed as described [12,48]. Briefly, total RNA (2 μg) was reverse transcribed using SuperScript II reverse transcriptase (Invitrogen). qRT-PCR was performed using the SYBR Green I PCR kit (Roche, Basel, Switzerland) and an iQ5 Real-Time PCR instrument (Bio-Rad, Hercules, CA, USA). Serial dilutions of GADPH (NM_002046) amplified cDNA were used as a control to generate standard curves and GAPDH from each sample was used as a loading control. Expression levels from control and treated samples were divided by control expression levels to deduce fold changes. Primer sequences are listed in Table S1.

### 4.5. Semi-Quantitative Real-Time Methylation-Specific PCR (qMSP)

qMSP experiments were performed and products were quantified according to the protocol described by Yan et al. [49]. Briefly, bisulfite-converted genomic DNA (0.5 μg) was subjected to real-time PCR with methylation specific primers (Table S1). qMSP reactions were performed using the SYBR Green I PCR kit (Roche) in an iQ5 Real-Time PCR instrument (Bio-Rad). Melting analyses were performed, followed by PCR reactions to ensure a specific amplicon was generated. *Col2A1* (NM_033150) was used as a loading control and to construct a standard curve. Serial dilutions of *Col2A1*-amplified bisulfite-converted DNA were used to generate the standard curve. The methylation percentage was calculated as: (Mean of target gene)/(Mean of *Col2A1*); fold change was calculated as: [Tumor (or treatment) methylation percentage]/[Control (or mock-treated) methylation percentage].

### 4.6. Methylation Sequencing

Genomic DNAs from control or *me_HR*-treated cells were bisulfite converted. *HIC1* methylation specific PCR (MSP) products were cloned and ten clones amplified from unmethylated or methylated primers were sequenced to confirm the methylation states. There was no amplification in MSC control using methylated primers. On the other hand, pyro-sequencing was used to detect the methylation states within *RassF1A* locus before and after targeted methylation.

### 4.7. AFM 

Cell stiffness was measured through AFM (Dimension Icon Bruker, Billerica, MA, USA) with DNP-10 triangular silicon nitride cantilevers. The nominal cantilever spring constant (k) and resonance frequency (f, in air) of the 205-mm-long cantilevers were 0.06 N/m and 12–24 kHz, respectively. A four-sided pyramidal tip with tip radius of 20 nm and half-open angle of the pyramidal face of θ ≈ 35° was used. Before performing every batch of measurements, the exact spring constant of each cantilever was calibrated through indentation on a silicon substrate and then determined using the thermal tune method. Cells were plated for longer than 12 h before each measurement, and measurements were performed at room temperature with tips dipped in serum-free media. A 20 × 20-μm^2^ area was initially scanned for the images around the cells, and cell stiffness was measured within an 8 × 8-μm^2^ area using a quantitative nanomechanical mapping mode [50]. In this scanning, all AFM images, such as height, peak force error, log Derjaguin–Muller–Toporov (DMT) modulus, and adhesion images were obtained. In this scanning mode, a force-separation curve, including tip loading and unloading curves, was recorded for every surface contact. Maximum loading force (peak force) was controlled quantitatively; therefore, the sample’s nanomechanical properties, such as elastic modulus, adhesion, and deformation, were obtained from the curves at each surface contact. Sixty-four force curves were deduced from each measured cell. Sneddon’s model was then fitted to each curve and the corresponding modulus was deduced and compared using Kruskal–Wallis tests and post-examined using Dunn’s multiple comparison. Peak force error is an AFM feedback signal similar to the deflection error in AFM contact mode or the amplitude error in AFM tapping mode. This signal correlated with the changes in height of the sample surface. Therefore, peak force error signal senses the height fluctuation of the sample. Compared with the height images, sample peak force error images depicted additional surface details and was selected to illustrate the changes within the cell surfaces in AFM scanning. Log DMT modulus was the logarithm of the derived sample elastic modulus based on the DMT model [51]. The tip radius and the sample stiffness, which were obtained from the unloading curves, were considered in this model.

### 4.8. In Vivo Tumorigenesis and Monitoring Tumor Growth

Three-week-old nude mice (Narl: ICR-Foxn1nu) were subcutaneously inoculated with 1 × 10^5^ cells. Tumor growth was monitored two months after inoculation by FMT4000 (Perkin Elmer, Billerica, MA, USA) using AngioSense tracking dye (Perkin Elmer). Tumors were surgically removed and subjected to immunohistochemical exams.

### 4.9. Immunohistochemistry

Tumor masses that were surgically removed were embedded in optimum cutting temperature media and sectioned into 10-μm sections on a cryostat (Leica, Buffalo Grove, IL, USA). Sections were stained with the indicated antibodies (HIC1 (Millipore), RassF1A (eBioscience), tubulin (GeneTex), and phalloidin (for F-actin, Invitrogen)), detected using fluorescein- or Texas red–conjugated anti-mouse or rabbit IgG (Vector Labs, Burlingame, CA, USA), followed by DAPI staining. Sections were also stained with hematoxylin and eosin (H&E; Vector Labs) for pathologic exams. For paraffin sections, staining followed the protocol described by Teng et al [12]. Tumor masses removed from nude mice inoculated with me_HR-transfected MSCs were paraffin-embedded and sectioned into 4-mm sections. Sections were stained with the indicated antibodies, and detection was performed using Vectastain (Vector Lab). Sections were also stained with H&E for pathologic exams.

### 4.10. Statistical Analyses

Paired Student’s *t* tests were used to test the methylation differences between tumors and non-tumors (*n* = 96). Spearman’s correlation tests were used to determine the correlation between *HIC1*, *RassF1A*, and tubulin gene expression in patient samples.

### 4.11. Human Subjects and Animal Care

Hepatic tumor samples were collected from Chang-Hua Christian Hospital according to their Institutional Review Board regulations (CCH IRB No.: 120504, 24 May 2012). The clinicopathological characteristics of 96 liver cancer patients are listed in Table S2. All mice were maintained, treated, and sacrificed in accordance with the protocols and regulations of Chung Cheng University Institutional Animal Care and Use Committee.

## Supplementary Materials

Supplementary Materials can be found at http://www.mdpi.com/1422-0067/19/10/2884/s1.

## Abbreviations

5-Aza	5-aza-2’-deoxycytidine
PCR	Polymerase chain reaction
AFM	Atomic Force Microscopy
MSP	Methylation specific PCR
