# Supplementary figures and images for: HIC1 and RassF1A Methylation Attenuates Tubulin Expression and Cell Stiffness in Cancer
Source: Int J Mol Sci. 2018 Sep 22;19(10):2884. doi: 10.3390/ijms19102884 (PMC6212922; doi:10.3390/ijms19102884)

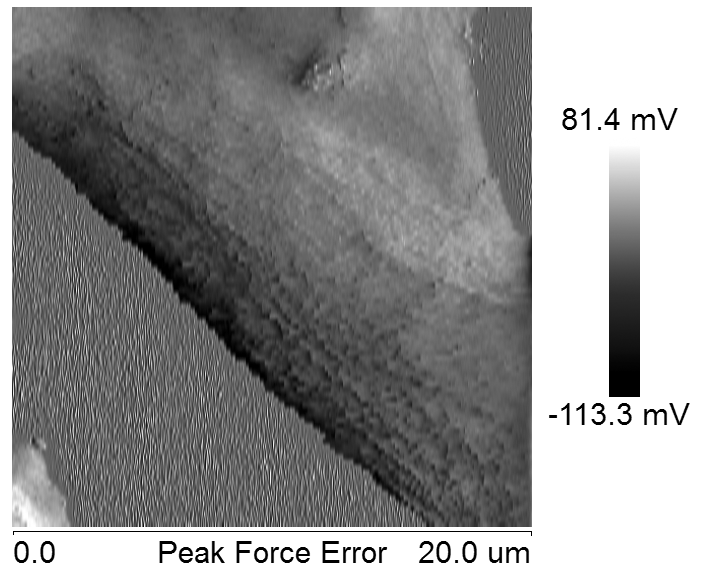

Supplement: Supplementary file 1 [file ijms-19-02884-s001.zip › Supplementary_Files/Chen_et_al_Supporting_Online_Material_File2_IJMS_d.gif]
