# Supplementary material for: HIC1 and RassF1A Methylation Attenuates Tubulin Expression and Cell Stiffness in Cancer
Source: Int J Mol Sci. 2018 Sep 22;19(10):2884. doi: 10.3390/ijms19102884 (PMC6212922; doi:10.3390/ijms19102884)
Supplement: Supplementary file 1 [file ijms-19-02884-s001.zip › Supplementary_Files/Chen_et_al_Supporting_Online_Material_File1_IJMS_d.doc]

**Supplemental Material**

*HIC1* and *RassF1A* methylation attenuates tubulin expression and cell stiffness in cancer

Chih-Cheng Chen, Bo-Ching He, Yao-Li Chen, Kuan-Der Lee, Chun-Hsin Tung, Chia-Chen Hsu, Ping-Yi Lin, Pei-Yi Chu, Yu-Wei Leu, Wei-En Fu and Shu-Huei Hsiao

Supplementary figures and text:

Supplementary Figure S1-3.

Supplementary Tables 1-4.

**Supplemental Figure S1. *me_HR* cells develop drug resistance.** (a) *me_HR* cells are less sensitive to cisplatin treatment. MSCs and derived *me_HR* cells were treated with designated concentrations of cisplatin, and cisplatin treatment caused cell death in MSCs. (b) Cell survival measured using MTT assays after cisplatin treatment.

Supplemental Figure S1

**Supplemental Figure S2.** **Clonal expansion of me_HR cells *in vivo*.** **(a)** Cytokeratin and CD133 were clonally detected in me_HR-derived tumor masses. me_HR cells were inoculated into immune-deficient mice and tumors that developed were surgically removed, cryosectioned, and immunostained with cytokeratin (red) and CD133 (green). Nuclei were stained with DAPI (blue). Clones expressing cytokeratin were observed, whereas CD133 was expressed in isolated cells. **(b)** NSE and CD133 were clonally detected in me_HR-derived tumor masses. me_HR cells were inoculated into immune-deficient mice and tumors that developed were surgically removed, cryosectioned, and immunostained with NSE (red) and CD133 (green). Nuclei are shown using DAPI staining (blue). Clones of NSE expression were observed whereas CD133 was expressed in isolated cells. **(c)** me_HR tumors expressed different surface markers. Tumors grown from me_HR cells were pathologically examined using the designated antibodies. Antibodies against vimentin detect the MSCs and CD133 antibodies detect potential stem cells. Antibodies against NSE and S-100 detect different epithelial markers.

Supplemental Figure S2

**Supplemental Figure S3.** **Immunostaining.** Immunostaining was performed on MSC (control), *me_HR* and 5-Aza treated *me_HR* cells with the designated antibodies.

Supplemental Figure S3

Supplemental Table 1: Primer Sequences

| Primer name | Gene | Sequence | Detection |
| --- | --- | --- | --- |
| H_GAPDH_RT_F | GAPDH (NM_002046) | CCCCTTCATTGACCTCAACTAGAT | RT PCR |
| H_GAPDH_RT_R |  | CGCTCCTGGAAGATGGTGA |  |
| H_TUBA1B_RT_F | TUBA1B  ( NM_006082.2) | GTCATTGATGAAGTTCGCACTGGCA | RT PCR |
| H_TUBA1B_RT_R |  | ACTGGTCAGCCAGCTTGCGAA |  |
| H_HIC1_RT_F | HIC1  (NM_006497) | GGACCATCGACCGTTTCTCT | RT PCR |
| H_HIC1_RT_R |  | GAGCTAAGGCACGAAGCAGT |  |
| H_HIC1_MSP_F |  | GGAGGACGGATTAGTAGGATAGATC | qMSP |
| H_ HIC1_MSP_R |  | AAAACCCACCTAAAAAACATATCG |  |
| H_ HIC1_MSP_M_F |  | ggTcgTtTTagataagagtgtgc | MSP  Sequence |
| H_ HIC1_MSP_M_R |  | CGAATCCAAAAAAAACGTAACTACG |  |
| H_ HIC1_MSP_U_F |  | GGTTGTTTTAGATAAGAGTGTGT | MSP  Sequence |
| H_ HIC1_MSP_U_R |  | CAAATCCAAAAAAAACATAACTACA |  |
| BR_137 | Col2A1  (NM_0033150) | TCTAACAATTATAAACTCCAACCACCAA | qMSP |
| BR_138 |  | GGGAAGATGGGATAGAAGGGAATAT |  |
| H_RassF1A_RT_F | RassF1A  (NM_007182) | TTGGAGACCCTGCAAACAGAACAG | MSP  Sequence |
| H_RassF1A_RT_R |  | GAAGCATTAAGGCACATGCTGTAC |  |
| H_RassF1A_MSP_ F |  | AGTTAGCGAAGTACGGGTTTAATC | qMSP |
| H_RassF1A_MSP_ R |  | ACCAACTACCGTATAAAATTACACG |  |
| H_RassF1A_ MSP_M _F |  | GAGAGCGCGTTTAGTTTCGTTTT | MSP  Sequence |
| H_RassF1A_MSP_M_R |  | ACCCGTACTTCGCTAACTTTAAACG |  |
| H_RassF1A_MSP_U_F |  | GAGAGTGTGTTTAGTTTTGTTTTT | MSP  Sequence |
| H_RassF1A_MSP_U_R |  | ACCCATACTTCACTAACTTTAAACA |  |
| H_RassF1A_Pyro _F |  | GGGTGTTAGTTTTAGTAGTTTAATGAGT | Bisulfite  Pyro-  sequence |
| H_RassF1A_Pyro _R |  | ACAATCCCTACACCCAAATTTC |  |
| H_RassF1A_Pyro _S1 |  | TTTAGAGTTTTTTTTAGGATTTAGA |  |
| H_RassF1A_Pyro _S2 |  | GTTAGTGTTTAGTTGGTTTTGG |  |
| H_RassF1A_Pyro _S2 |  | GTAGTTTAATGAGTTTAGGTTT |  |
|  |  |  |  |

Supplemental Table 2: Clinicopathological characteristics of 96 patients with hepacellular carcinoma

|  |  | Patients  *N* = 96 (%) |
| --- | --- | --- |
| Age | Mean (SD) | 67 (10) |
| Gender | Male | 71 (74) |
|  | Female | 25 (26) |
| Tumor Size (cm) | Mean (SD) | 5.1 (3.5) |
| Stage | I | 20 (21) |
|  | II | 63 (65) |
|  | III | 13 (14) |
| Histology grade | I | 4 (4) |
|  | II | 45 (47) |
|  | III | 47 (49) |
| *HIC1* | Hypomethylation | 65 (68) |
|  | Hypermethylation | 31 (32) |
| *RassF1A* | Hypomethylation | 13 (14) |
|  | Hypermethylation | 86 (86) |

Supplemental Table 3: *HIC1* gene methylation status in hepatocellular carcinoma patients according to clinicopathological characteristics

|  |  | Hypo Methylation | Hyper Methylation | *P* value |
| --- | --- | --- | --- | --- |
|  |  | *n =* 65 (%) | *n* = 31 (%) |  |
| Age | Mean (SD) | 67 (10) | 67 (8) | 0.987^a^ |
| Gender | Male | 51 (78.5) | 20 (64.5) | 0.145 |
|  | Female | 14 (21.5) | 11 (35.5) |  |
| Tumor size (cm) | Mean (SD) | 5.4 (3.8) | 4.6 (3.2) | 0.686^a^ |
| Stage | I | 12 (18.5) | 8 (25.8) | 0.276 |
|  | II | 43 (66.2) | 20 (64.5) |  |
|  | III | 10 (15.4) | 3 (9.7) |  |
| Grade | Well | 2 (3.1) | 2 (6.5) | 0.694 |
|  | Moderate | 30 (46.2) | 15 (48.4) |  |
|  | Poor | 33 (50.8) | 14 (45.2) |  |

^a^ Mann-Whitney U tests; * *P* < 0.05

Supplemental Table 4: *RassF1A* gene methylation status in hepatocellular carcinoma patients according to clinicopathological characteristics

|  |  | Hypo Methylation | Hyper Methylation | *P* value |
| --- | --- | --- | --- | --- |
|  |  | *n =* 13 (%) | *n =* 86 (%) |  |
| Age | Mean (SD) | 67 (7) | 67 (10) | 0.889^a^ |
| Gender | Male | 8 (61.5) | 63 (75.9) | 0.272 |
|  | Female | 5 (38.5) | 20 (24.1) |  |
| Tumor size (cm) | Mean (SD) | 5.0 (2.7) | 5.1 (3.6) | 0.406^a^ |
| Stage | I | 4 (30.8) | 16 (19.3) | 0.274 |
|  | II | 6 (46.2) | 57 (68.7) |  |
|  | III | 3 (23.1) | 10 (12.0) |  |
|  | IV | 0 (0) | 1 (1.2) |  |
| Grade | Well | 0 (0) | 4 (4.8) | 0.512 |
|  | Moderate | 5 (38.5) | 40 (48.2) |  |
|  | Poor | 8 (61.5) | 39 (47.0) |  |

^a^ Mann-Whitney U tests; * *P* < 0.05
